# The social life of viruses: a queer sort of science

**DOI:** 10.1007/s40656-026-00772-z

**Published:** 2026-10-15

**Authors:** Timothy Gitzen

**Affiliations:** Department of Anthropology, Wake Forest University, Winston-Salem, NC USA

**Keywords:** Microbes, Horizontal gene transfer, Queer theory, Temporality, Evolution

## Abstract

A burgeoning microbiology field, sociovirology is the study of how viruses interact with one another and the effects such interactions have on social evolution. Challenging dominant narratives that viruses are individualistic pathogens, sociovirologists explore the social interaction of viruses through the language of cooperation, altruism, and cheating, social concepts with biological definitions. Viruses cannot self-replicate—they must rely on living cells to reproduce—and thus are commonly believed to be biologically nonliving. By focusing on virus-virus interaction and evolution, sociovirologists are displacing reproduction as a chief engine of aliveness and queering biology and the concept of life. I contend that in sociovirological publications, the aliveness of viruses is indexed through sociovirologists’ use of language reserved for the interactions and social behaviors of life-forms alongside viruses’ ability to evolve. This paper illustrates that by attending to social behavior and evolution, sociovirologists are proffering novel approaches to both viruses and the concept of life that displaces individual organism reproduction and vertical inheritance as normative of life with species reproduction and species evolution as normative *for viruses* even if nonnormative for biological understandings of life. Viruses are alive because, as species, they evolve despite an individual viral particle’s fate.

## Introduction

In their capaciously titled book *What is Life?* Lynn Margulis and Dorian Sagan (2000, p. 14) proffer life as a verb, a doing: “It repairs, maintains, re-created, and outdoes itself.” For Margulis and Sagan (2000, p. 17), life—both as a noun and a verb—hinges on autopoiesis, or the continued production of itself through the ability to metabolize, whereby “without autopoietic behavior, organic beings do not self-maintain—they are not alive.” Even replication is not as “fundamental a characteristic of life as is autopoiesis” given that there are countless examples of sterile animals, like mules, that do not “replicate” but are still autopoietic because they metabolize (Margulis & Sagan, 2000, pl 18). And so, as Margulis and Sagan argue, viruses are not alive because they are not autopoietic. Even if autopoiesis is the chief engine of life, Margulis and Sagan (2000, p. 18) write that viruses rely on “an autopoietic entity,” that “viruses reproduce within their hosts,” and as such, “without an autopoietic organic being, a biological virus is a mere mixture of chemicals.” In other words, while autopoiesis marks organic entities as living, viruses are considered nonliving entities because their replication/reproduction is dependent on living, autopoietic entities. Their replication/reproduction mechanism thus deems viruses as nonliving and frame, more generally, reproduction/replication as individualistic and unassisted by other entities or technologies.

This litmus test of life, both the emphasis on autopoietic behavior and reproduction/replication, excludes certain bodies from consideration and is rooted in cisgender, heteronormative, able-bodied structures and discourses (Malatino, 2019).^1^ That a body requires external support or assistance by way of medications, technology, and even infrastructure would deem these bodies nonliving by strict autopoietic standards. Furthermore, that viruses rely on living entities to replicate implies that assisted replication (or reproduction) does not count as “actual” or “true” reproduction.^2^ When scaled up, this implies that reproduction that makes use of reproductive assistive technologies (such as IVF) is somehow less than “true” than natural forms of heteronormative reproduction. For Margulis and Sagan (2000) to hang the banner of life around autopoiesis thus ignores our augmented, cyborg human reality: who among us humans can say to be wholly individual and devoid of augmented assistance (Haraway, 1991)? Indeed, as Sagan (2013) later observes, our very lively existence is reliant on other microbial lifeforms and even viruses in our guts, brains, and even genome. In fact, mammalian reproduction evolved due to endogenous retroviruses: the development of placenta is due to “nonliving” viruses (Moelling & Broecker, 2019). But alas, for Margulis and Sagan (2000, p. 18), the virus is but “a mere mixture of chemicals.”

*A mere mixture of chemicals.* Despite the fact that we are all a mixture of chemicals—from humans and microbes to rocks and the wood used to build my father’s house—to be *merely* anything implies that viruses are unimportant, unremarkable, just so. Quite the contrary, many in the field of virology would argue: viruses are extraordinary if only because they continue to surprise us with every twist and turn of viral discoveries. A burgeoning field of virology challenges the dictum that viruses are merely anything, let alone that autopoiesis and reproduction are the chief engines of life by focusing on viral evolution.

Margulis and Sagan (2000, p. 19) argue that “for a population to evolve, its members must reproduce” and that “before any organic being can reproduce, it must first self-maintain,” but this new field, *sociovirology*, has a different view of evolution and viruses. For sociovirologists, viruses have complex social lives *between themselves* that impact their evolution, for even if viruses biologically rely on cells to reproduce, viruses still mutate and evolve. In earlier studies, some scientists argued for the preclusion of viruses from the tree of life because, for the most part, all their functions rely on the cell: they do not metabolize or self-replicate, and even their evolution, these authors argue, is reliant on cells (Moreira & López-García, 2009). While viruses do evolve within cells, Eugene Koonin and colleagues (2022, p. 917) contend that viruses have their own genomes and “evolutionary trajectories” as they are “locked in arms races with their [cellular] hosts.”^3^ Sociovirology similarly hinges on the ability of viruses to evolve both in relation to cells/hosts and in relation to other viruses. For sociovirologists, I argue, that ability to evolve is the marker of viral aliveness, *not reproduction* or even autopoiesis. Viruses live regardless of cellular hosts because they evolve, and they evolve because viruses interact with and behave in relation to each other. In this way, sociovirology is a queer sort of science, displacing the primacy of reproduction as the key marker of life.

This article is an examination of sociovirology and the field’s exploration of the social life of viruses. I aim to centralize the question of viruses’ aliveness as not epiphenomenal to their behavior or evolution, but quintessential to understanding both given that their social behavior and evolution challenges a conception of life that centralizes reproduction. Even though Margulis and Sagan (2000) argue that autopoiesis is the first marker of life, when describing viruses, the question of aliveness reverts to reproduction. It is seemingly inescapable: to tell the story of viruses is to tell of their inability to self-replicate or reproduce, a very human, downright heteronormative concern. Sociovirology tells a different story, a queerer story that begins not with reproduction, but social behavior. In rather queer fashion, sociovirologists sidestep not only reproduction but the engine of reproduction—the cell—and focus primarily on the interactions between the seemingly unalive “mixture of chemicals.” Doing so they demonstrate the profound sociality that exists between viruses themselves and taken even further, that said sociality influences viral behavior and viral evolution.

I argue that sociovirology queers biology and even life by centralizing virus-virus interaction and evolution as implied markers of life. While the sociovirological studies I discuss herein are less concerned with defining life or arguing for viruses’ living status—it is either implied or stated as an off-hand comment, as evidenced in the epigraph—I contend that the social implication of their work redirects attention away from reproduction and towards viral behavior and evolution not predicated on self-replication. The heteronormative approach to life upholds reproduction as its central hallmark, but as I demonstrate with sociovirology, viruses are queer biological entities that challenge the contours of life by living *despite* reproduction. This is not to say that viruses do not replicate or reproduce; they do within cells, and this is paramount to their continued existence. But viruses can rely on cells to replicate and independently exist as life-forms simultaneously. My attention is on the ways sociovirology focuses on the latter, on the quality of viral existence through behavior and evolution.

This piece moves through three parts. In the first part I unfurrow the connections of life and reproduction through queer theory, attending to the heteronormative impetus to centralize reproduction as the hallmark characteristic of classifying biological entities as living. I then move into sociovirology and interrogate the language used by sociovirologists and science writers to describe viruses. This language has historically been used to talk about biologically living entities and so by exploring the uses of such language, I contend sociovirologists are implying the aliveness of viruses. This dovetails into the third part where I explore the ways sociovirologists connect virus-virus interaction to evolution, a chief characteristic of life. In this part I extrapolate the importance of temporality in evolution to show the queer ways viruses challenge normative temporal formations. I conclude by returning to the question of life and how sociovirology proffers a queer understanding of life that not only includes viruses but redirects attention away from reproduction and towards social interaction and evolution.

A note on methodology. While I primarily rely on the works written by self-proclaimed sociovirologists and their biologist commentators, I also turn to popular science writers that discuss the burgeoning field of sociovirology and its key mechanisms of collaboration, altruism, and cheating. I am less concerned with doing a strict analysis of scientific papers than I am about interrogating the field of sociovirology, and that means turning to both scientific and popular discourse of the field. In particular, attention to science reporting on sociovirology not only demonstrates the growing popularity of the field but it also articulates perhaps more directly (and forcefully) the stakes of the field beyond the confines of virology. Stated alternatively, I turn to popular science writing to augment my analysis of scientific papers as a way to contextualize the field and language of sociovirology within broader social discourse around viruses and science. As such, science writing provides necessary depth to the field of sociovirology not wholly captured in scientific papers alone.^4^ I should also note that science writing relies not only on the papers scientists write, but interviews with the scientists themselves and thus can summarize fields, mechanisms, studies, and processes in more accessible language. This is important for any queer feminist methodology that seeks to decentralize knowledge production and bring multiple kinds of knowledge into conversation with one another, including not only scientific papers and science writing, but queer theory as well.^5^

## Displacing reproduction, queering life

In a 2008 piece for *Scientific American* entitled “Are Viruses Alive?” virologist Luis Villarreal briefly sums up the history and placement of viruses in biology:

[T]he scientific community has repeatedly changed its collective mind over what viruses are. First seen as poisons, then as life-forms, then biological chemicals, viruses today are thought of as being in a gray area between living and nonliving: they cannot replicate on their own but can do so in truly living cells and can also affect the behavior of their hosts profoundly. The categorization of viruses as nonliving during much of the modern era of biological science has had an unintended consequence: it has led most researchers to ignore viruses in the study of evolution. Finally, however, scientists are beginning to appreciate viruses as fundamental players in the history of life.

Villarreal does not give us an answer to his question because, as he notes, it depends on what we define as “life.” He admits that most definitions of life involve some admission of self-replication and autopoiesis. Viruses, by this definition, are thus not alive. For Villarreal, the real issue at hand is not actually if viruses are alive or not, but the role viruses play in the evolution of life itself, a role that historically had been overlooked—and still ignored by many—in favor of a straight-forward narrative that viruses are nonliving parasites and pathogens. Patrick Forterre (2016, p. 101) makes a similar claim, arguing that “we should probably modify the meaning of common concepts, such as life or organisms, when applied to biology, in order to make them useful for an objective description of nature, and introduce new concepts (such as the virocell concept) to prevent some of the ambiguities inherent to the current paradigms.”^6^

Villarreal is not alone in shifting attention to the importance of viruses in what is commonly referred to as the “Tree of Life” (Bandea, 2009; Forterre, 2010; Pennisi, 2013), a phrase that microbiologists continue to use to describe the evolution of life from three main branches: bacteria, archaea, and eukaryote. In this tree of life, viruses exist outside the confines of these branches and/or play a direct role in the evolution of all three branches. As Villarreal and Guenther Witzany (2010, p. 698) contend, “it must be considered that viruses have played and still play crucial evolutionary roles and are essential agents within the tree of life.” Challenging those virologists who exclude viruses from the tree of life because of their genetic diversity, Villarreal and Witzany (2010, p. 706) conclude that “the very genetic volatility of viruses, used as argument to exclude them from the tree of life must now be considered as an essential *precondition* for life” (emphasis in original). Dennis Bamford (2003, p. 234) makes an analogous claim, noting that “with the observation that cellular life is intimately linked with the world of viruses, and possibly always has been, it seems that viruses may form lineages that extend from the root to all branches in the tree of life.”^7^ Given the genetic diversity of viruses and the fact that viruses weave in and out of the tree of life, “it will never be possible to draw a universal tree of viruses analogous to the tree of the LUCA [last universal cellular ancestor]” (Forterre & Prangishvili, 2009, p. 466).

Anthropologist Stefan Helmreich (2009, p. 87) demonstrates that lateral or horizontal gene transfer complicates further the tree of life. As he writes, “lateral gene transfer in microbes places in jeopardy the vertical inheritance needed to root the tree of life.” Lateral gene transfer is the transfer of genetic material between organisms that are not classified as parent and offspring (Irwin et al., 2022). For Helmreich (2009, p. 87), an anthropological approach demonstrates that “a culturally reinforced biogenetic understanding of relatedness pressed researchers into phylogenies based on genes, splicing a genetic logic into the arborescent image of the family tree […whereby] rather than pointing toward a common ancestor…this focus fragmented the very object of inquiry.”^8^ Complicating this arboreal metaphor even further is that viruses can act as vectors for lateral gene transfers, including gene transfer in eukaryotes, the classificatory domain that includes both fungi and animals. Viruses can laterally transfer genes to other viruses, especially when two viruses infect the same host, but viruses can also transfer their genes to the host through viral endogenization, a process where viral genetic material integrates into the host’s genome. It is estimated that roughly 8% of the human genome is comprised of endogenous retroviruses. These are rather queer practices as they sidestep direct descent reproduction and yet still manage to make relations out of those viruses and hosts with which genes are laterally transferred (see Bradshaw, 2023).

Queer theory is a useful tool for explicating not only the practices of lateral gene transfer, but the queerness of viruses more broadly. As queer theorist Neel Ahuja (2015, p. 369) writes of queer theory,

I seek a critical discourse that inquires more deeply into the micropolitics of reproduction and extinction, where racial divisions of climate change in the intimate scales of contact between human social forms and ecologies of production and waste…a queer-theoretical response to late-carbon liberalism might involve thinking reproduction as an interspecies entry point into entangled forms of violence—forms often distorted by moralizing and universalizing figures of the parasite.

Central to Ahuja’s queer theory is a critique of heteronormative reproduction, that a queer response to the alterability of our bodies within late liberalism (Murphy, 2017) is to rethink reproduction through interspecies entanglements and relations. Indeed, as queer feminist theorist Stephanie Clare (2019, p. 54) writes in their sideways reading of epigenetics, and following renowned feminist philosopher of science and scientist Evelyn Fox Keller (2010), “there is no reason to prioritize birth in our understanding of the biological: ‘development is lifelong, and so is its plasticity.’” To interweave other interspecies entanglements along the longue durée of evolutionary time is to attend to a queer theory of more-than-human enmeshments that permeate our ecologies.

In sociologist David Griffiths’ (2015, p. 36) treatise on queer theory and lichens, he contends that “lichens and other symbioses suggest a queer ecological perspective that could go some way toward denaturalizing the primacy of heterosexuality and sexual reproduction in defining legitimating bodies, practices and communities.” To do this, Griffiths details how a symbiotic view of life displaces the developmental and genetic individuality of humans, in addition to other biological life, so that the microbial interconnections that form down to the genetic and DNA level are always more-than-human. To focus on the “queer connections and cominglings,” like Griffiths does, is to “decenter heterosexual biological reproduction and vertical inheritance” (Griffiths, 2015, p. 38) and thus displace the primacy of arboreal metaphors for life and the ways vertical inheritance and reproduction are centralized in biological and even social understandings of life. Viruses thus fit within this queer theory for lichens given that both viruses and lichen rely on communities of micro- and macro-organisms to sustain themselves, thereby shelving reproduction and centering the lateral connections that form across species. Viral particles may exist in and of themselves—as does the unicellular microbe—but their liveliness arises in their sociality, their “queer connections and cominglings” with other viruses, microbes, and cells. But as Griffiths (2015, p. 41) observes of viruses like HIV, “people living with viral infections [are seen] as dangerous intruders themselves…that must be eradicated or kept out of the political nation state.” I make a similar observation elsewhere about viral infections in South Korea, and akin to Griffiths, I suggest that interpreting the human body as a “human-virus hybrid” not only attunes to the biological reality of the human body but can work to challenge the pathogenic narrative of viruses and, more specifically, the threat-making capacity attached to people living with HIV/AIDS and other viral infections (Gitzen, 2023). To focus on the social life of viruses is to thus explicate the “queer connections and cominglings” of viruses as indexical of life itself. Doing so, I suggest, indexes alternative methods of relation-making that extend beyond the vertical inheritance of (sexual) reproduction: viruses are making kin, they are a “complex generator of life” that entangle various life-forms in their sociality (Kirksey 2019, p. 208).

Queer ecology is useful in explicating the social life of viruses. Philosopher Timothy Morton (2010, p. 275), inspired by Darwin, argues that “life-forms are liquid” and that a queer ecological approach must account for this liquidity: “I propose that life-forms constitute a *mesh*, a nontotalizable, open-ended concatenation of interrelations that blur and confound boundaries at practically any level.” We again return to Griffiths’ (2015) proposition that we are all lichen for our symbiotic and symbiogenesis existence, and notably, that such lichen existence displaces heterosexual biological reproduction as the chief engine of life and life-forms. For as Morton (2010, p. 277) contends, “queer ecology would go to the end and show how beings exist precisely because they are nothing but relationality, deep down—for the love of matter.” Not only are life-forms caught in the meshes of ecological interconnections, but our relatedness, as I explore below, is not solely predicated on reproduction and vertical inheritance; horizontal gene transfer also enables a relatedness between species and life-forms. As anthropologist Eben Kirksey (2019, p. 214) writes of microbes, including the *Wolbachia* bacteria and HIV, they are “helping humans forge new promiscuous and convivial associations—breaking down filial divisions along lines of race, class, and nationality to generate new queer kinship networks.”

Challenging heterosexual biological reproduction and thus sexual intercourse is to simultaneously propose alternative modes of kin and community making. Clare (2019, p. 53) makes a similar observation in their work on epigenetics, contending that epigenetics “can help make clear how communities of belonging—not just those considered family—become embodied.” They explain, for instance, how epigenetics’ “focus on the expression of DNA seems to problematically recenter genes, [but] this centering can also be strategic…reimagining biological relatedness in a way that displaces the centrality of heterosexuality and dimorphic gender relations, which have been naturalized in prevalent understandings of that relatedness” (Clare, 2019, pp. 54–55).^9^ Queer feminist theorist Tarsh Bates (2019, p. 27) similarly writes of *C. albicans*, that it “literally grasps its environment,” for “communities form and dissolve as communication is ingested, digested and excreted.” No biogenetic theory of kin selection can thus explain this kind of “microbial sociality.” Bates (2019, pp. 30–31) admits that “fitness is measured by population growth, firmly placing reproductivity and fecundity as the central poles of kin selection.” Yet as Bates (2019) demonstrates with *C. albicans*, sexual reproduction is a rather uncommon practice among living organisms; they reproduce or replicate through other, non-sexual means (see also Subramaniam & Bartlett, 2023).^10^

What would it mean to displace reproduction and vertical inheritance as the hallmarks of life and what constitutes a life-form? It would mean, at first, to take lateral gene transfer seriously as a form of queer relation-making.^11^ As described above, lateral gene transfer enables microbes, viruses included, to exchange genetic material within and between species, as microbes can also exchange genetic materials with hosts. This biological mechanism does not create genetic progeny in the way that reproduction and vertical inheritance does and thus eschews a heteronormative reproductive telos. Queer theory is rich in its critique of heteronormative reproduction as central to social life and normative bodies (e.g., Edelman, 2004; Halberstam, 2005), but it also attends to the vertical and horizontal movements of subjects, whereby children mature, grow *up*, and are descendants of vertical inheritance. Queer subjects, including queer children, “grow sideways,” queer theorist Kathryn Bond Stockton (2009) contends, as movements are not predicated on a maturation narrative or on growing up in some linear fashion; rather, queer subjects move laterally within the moment, branching in every which direction but up as a way to breathe, survive, and even thrive (Gitzen, 2025a). Similarly, lateral gene transfer is a form of sideways growth and change not beholden to the rigidity and heteronormativity of vertical inheritance rooted within reproduction. That there are different ways genes are laterally transferred—through processes of transformation, conjunction, or transduction—illustrate that there is not one, centralized way to transfer genes but multiple, non-vertical methods outside of reproduction. As a queer method, lateral gene transfer does not mark life-forms any less alive because it eschews vertical inheritance and reproduction/replication. Instead, it resituates what makes life-forms alive, or in biological language, it reassesses the organismality (what makes an organism an organism) of the life-form.

The second way to displace reproduction and vertical inheritance is to displace the temporality associated with reproduction and inheritance by refocusing attention on the longue durée of evolutionary time. Evolutionary theory, in Morton’s (2010, p. 275) estimation, “is antiessentialist in that it abolishes rigid boundaries between and within species.” This is, for Morton (2010, p. 275), a queer sort of science given that “queer theory is a nonessentialist view of gender and sexuality.”^12^ While I explore this in greater detail below, evolutionary time is less concerned with the “chronormativity” of the individual organism’s reproduction (Freeman, 2010) and instead charts a longer course across species as the species moves through generations. To think evolutionarily or generationally is to displace the importance of immediate reproduction and replication for the betterment or continued existence and evolution of the species. In other words, it matters less if the individual can replicate or reproduce if the species perpetuates *over time*.

## A social understanding of viruses

In late March 2022, roughly 25 microbiologists gathered at St. John’s College at Oxford University to discuss the burgeoning field of sociovirology, what some also call the social life of viruses. This was the first of such a meeting of the minds, the second taking place in 2024 and the third scheduled for some time in 2026. Organized by Asher Leeks and Sam Díaz-Muñoz, two of the leading scholars in sociovirology, the conferences aim to bring together discussions of social phage behaviors, minority variants, defective interfering genomes, multipartite viruses, and evolutionary theory together to discuss the ways viruses interact with one another and how such interactions impact evolution and host interaction (Leeks, 2025).^13^ Leeks and colleagues (2023, p. 1552) along with Díaz-Muñoz and colleagues (2017) contend that what we would call “sociovirology” is, simply put, a field of study that attends to “virus-virus interactions” or the “social interactions” of viruses. For both sets of scholars, such social interactions drive the evolution of viruses and their social traits.

Science writer Carl Zimmer (2024) writes popularly for Quanta Magazine of the emergence of this field of virology as a pushback against the commonly held assumption that viruses are simple. As Zimmer writes:

If you think of viruses as isolated packages of genes, it would be absurd to imagine them having a social life. But…a new school of like-minded virologists don’t think it’s absurd at all. In recent decades, they have discovered some strange features of viruses that don’t make sense if viruses are lonely particles. They instead are uncovering a marvelously complex social world of viruses. These sociovirologists…believe that viruses make sense only as members of a community.

Zimmer draws our attention to the commonly held assumption about viruses, namely that they are “isolated packages of genes.” But he then quickly challenges this assumption by positing that viruses have social lives, for viruses are behaving in ways that do not make sense if thought of only as “isolated packages of genes.” In short, the central claim of sociovirology is that viruses are not stand-alone, asocial entities but are instead intensely social organisms that interact with one another through acts of collaboration, altruism, and cheating.

Collaboration, altruism, and cheating are decidedly social traits—sociological language used to often describe human behavior, but in biology, they also have very pointed definitions to describe the behavior of animals and other living organisms. It is first important to define what biologists mean by “social” or “social interaction. Neurobiologists Peggy Mason and Haozhe Shan (2017, p. 3) proffer a new, broader definition of sociality, namely “any significant deviation from behaviour exhibited by an individual when that individual is within a group of two or more.” Key to Mason and Shan’s (2017) explication of the social is that sociality is a domain of the living, and so while living organisms may form relations with nonliving entities—the rat’s relationship to the cage—sociality is predicated on at least two living organisms in relation to one another. This is important when considering that sociovirologists mobilize a similar broad definition of sociality. Díaz-Muñoz and colleagues (2017, p. 437) define social interaction as when “the traits of one individual influence the fitness of another individual” and as such, “evolutionary analysis of social interactions relies fundamentally on natural selection.” A virus must cause changes in the other’s behavior and fitness for these viruses to be social, where fitness is defined as the ability to survive and reproduce. Important to note is that sociality is an evolutionary mechanism, one that changes over long periods of evolutionary time.

Understanding the biological and sociovirological definition of the social enables a more situated discussion of cooperation, altruism, and cheating. Cooperative traits are those that benefit others and “will only be selected for by natural selection if they provide a fitness benefit to the individual performing them” (Díaz-Muñoz et al., 2017, p. 437), meaning that they cannot evolve only for the sake of the population. Leeks and colleagues (2021, p. 6928), however, offer a different understanding of viral cooperation, defining cooperating as “performing an act or trait that is maintained by natural selection because it benefits another individual […] it should reduce the relative fitness of the individual performing the cooperation, and hence be selected against.” Unlike Díaz-Muñoz and colleagues (2017, p. 437) definition, cooperation hinges on the benefit to another individual viral particle—cooperation is not about individual fitness but about the fitness of others. As Leeks and colleagues (2021, 6982) note of their definition, “the evolutionary definition of cooperation that we use here differs from the way that cooperation is often used in virology, because our definition excludes viral traits that benefit other individuals *as a by-product*, but that are maintained by natural selection solely due to benefits to the trait-holder” (emphasis added). In other words, the benefit to other individual viral particles is quintessential to how viruses behave and socially evolve.

In this way, viruses that cooperate are not necessarily concerned with their own individual survival. Some viruses are even altruistic. Leeks and West (2019, p. 910) write that altruism is when a behavior or trait “is costly to perform and provides a benefit to others,” namely the species or population. Science writer Elie Dolgin (2019) profiles microbiologist Joe Bondy-Denomy and colleagues’ work on phages (viruses that infect bacteria), explaining that the teams “watched as viruses bombarded bacteria with specialized proteins designed to break down the cells’ CRISPR-based immune defences.” CRISPR, or clustered regularly interspaced short palindromic repeats, is the natural defense of bacteria against viruses carried out by “remembering” and destroying viruses. Dolgin (2019) continues: “the initial bombardment paved the way for others to conquer the microbial foe.” As Bondy-Denomy himself notes of this example, “those phages had to be there, and to die, and produce anti-CRISPRs before another phage could come along and succeed” (Bondy-Demony in Dolgin, 2019). In other words, some individual viral particles seem to exist solely for the benefit of other viral particles, that they “die” so that others may “live.” Among animals, for instance, bees and ants are often known for their altruistic behavior given that the vast majority work with no chance for reproduction.

At the other end of the viral spectrum are cheaters. Cheating is “a trait that is beneficial to a cheat and costly to a cooperator in terms of inclusive fitness…when these benefits and costs arise from the actor directing a cooperative behavior toward the cheat, rather than the intended recipient” (Ghoul et al., 2014, p. 319). Key here is that the benefit lies with the cheating virus and not the viruses that were intended to receive the benefit. As Leeks and colleagues (2021, p. 6928) write, “cheating can be thought of as a special form of parasitism, in which the parasite exploits a social trait, benefitting from the trait in the same way that the trait has evolved to be used by cooperators. Cheating, therefore, exerts specific selection pressures that can select against cooperation, and/or shape the evolution of cooperative traits.” Again, the benefit is aimed towards the cooperator, but the cheat takes advantage of the situation to gain the benefit instead. Writing of altruism and cheating in viruses, science writer Viviane Callier (2019) explains that “in an evenly mixed system, altruistic viruses fall victim to ‘cheaters’ that take advantage of their sacrifices.” Callier provides the example of vesicular stomatitis virus (VSV), a less dangerous member of the rabies family. VSV causes its mammalian host to produce interferons, “the signaling proteins that raise neighboring cells’ antiviral defenses and interfere with viral replication” (Callier, 2019). The wild-version of VSV has evolved so that it can suppress the host’s immune system, but it does so by reproducing more slowly. This version of VSV will thrive unless a cheater variant emerges. Cheats cannot suppress their host’s immune defenses and instead stimulates the production of interferons. However, they still take advantage of the lower immune response of nearby VSV particles that suppress interferon production. As Callier (2019) writes, “because the cheaters don’t pay the reproductive cost of interferon suppression, they can outcompete the wild-type virus in the short term.” The wild-type VSV thus classifies as altruistic in this cheating scenario.

The social biological language of cooperation, altruism, and cheating allows sociovirologists to focus less on the reproduction, or lack thereof, of viruses and instead on their social behaviors. Doing so, I argue, indexes the living quality of viruses. Indeed, for some sociovirologists, a virus’ living status is an already settled matter, as evidenced in the epigraph. This mention of living organisms in the epigraph is an interesting way for Leeks and colleagues to acknowledge viruses living status, for rather than state point blank “viruses are living organisms,” they instead lump viruses into the category of “living organisms.” Part of what the authors are calling for in this piece, particularly in relation to the phrase “living organism,” is a theoretical discussion into what constitutes a viral organism: “viral organismality is an area where careful theory would be useful, linking the evolutionary idea of organismality to viral biology” (Leeks et al., 2023, p. 1559). The invocation of organismality, or how to understand organisms as organisms, is yet another nod towards the aliveness of viruses given that biological organisms are definitionally alive. Leeks and colleagues (2023, p. 1559) conclude of viral organismality that “in an evolutionary sense, the presence of both conflict and cooperation [in viruses] means that viral infections are more analogous to societies than to organisms.” Yet even here, a society is comprised of living organisms.^14^ Perhaps, then, a more apt understanding of the virus is not an individual life-form but a “living process” (Dupré and Guttinger 2016), a process that spans both temporality and species (Gitzen, 2025b).

## In the time of evolution

The social language of cooperation, altruism, and cheating utilized in sociovirology hinges on the crucial role social evolution plays in sociovirology. As Díaz-Muñoz and colleagues (2017, p. 437) write, a social evolution framework “can clarify unexplained phenomena in virus-virus interaction by identifying evolved social traits, their genetic and mechanistic basis, and the selective pressures underlying these traits.” I wish to stay with the basic premise that virus-virus interaction is best explained through social evolution theory and that such viral interactions impact evolution and host interactions. For instance, Díaz-Muñoz and colleagues (2017, p. 437) detail the interplay between cooperation and evolution, noting that.

cooperation can be evolutionary favored…when it is directed toward genetically identical individuals or relatives. From an evolutionary perspective, helping a genetically identical individual reproduce is the same as reproducing yourself. By extension, helping relatives reproduce provides a fitness benefit to the actor, as relatives share a fraction of the actor’s genes. This process by which individuals increase their fitness by helping relatives reproduce is termed kin selection.

In this definition, kin selection relies on helping relatives reproduce, which thereby increases the fitness of the cooperating individual. To be kin is to be genetically relative to the individual while also being enough of a relative to help reproduce for it is equated to self-reproduction. Leeks and colleagues (2021, p. 6928) extrapolate the issue of viral cooperation and evolution by explaining that cooperation will happen, first, if such practice benefits the “individual performing the cooperation.” Cooperation will also happen, as noted above, if such practice benefits “other individuals that carry the cooperative gene, even if it is costly overall to the cooperative individual.” This, Leeks and colleagues (2021, p. 6928) argue, is termed “kin selection, because the easiest and most common way for individuals to carry the same genes is through common descent. By helping a close relative reproduce, an individual is still passing on its genes to the next generation, just indirectly.” Leeks and West (2019, p. 910) make an analogous claim of altruism, namely that the “cost and benefit” of altruism “are defined in terms of evolutionary fitness—the number of offspring produced. Explaining altruism is a fundamental problem for evolutionary biology.” Akin to Díaz-Muñoz and colleagues (2017), Leeks and West (2019, p. 910) similarly point towards relatedness as a lynchpin in the social evolution of viruses, noting that “relatedness is a measure of genetic similarity (kinship) that essentially captures the likelihood that they would share a gene for altruism.”

While I recognize the intersecting histories of Charles Darwin and Henry Lewis Morgan in the nineteenth century, both fathers of kinship in their own rights (Service et al., 1981; van der Grijp, 1997), a kinship or relatedness of viruses is a rather queer contraption. My discussion of lateral or horizontal gene transfer above as a form of queer relation-making at first recognizes that biologically, horizontal gene transfer can increase local “relatedness” between two organisms—that their genetic material has greater similarity—and thus affect kin selection in a complementary manner (Mc Ginty et al., 2013). In this definition, horizontal gene transfer and kin selection are separate, though it is telling that horizontal gene transfer can increase the *relatedness* of organisms. I am reminded of Griffiths’ (2015, p. 37) insistence that biological and social language must be interpreted alongside one another—that they are indexical of one another, as the anthropology of kinship and “relatedness” has shown. Anthropologist Janet Carsten (2000,p. 4), for instance, mobilizes relatedness “to suspend a particular set of assumptions about what is entailed by the terms social and biological…a move away from a pre-given analytic opposition between the biological and the social on which much anthropological study of kinship has rested.” Carsten’s goal is to provide a more capacious understanding of how, locally, cultures map and navigate relations, what anthropologists have historically called kinship. While horizontal gene transfer may not biologically make kin out of organisms, they do form relations, relations that challenge and queer the primacy of vertical inheritance, reproduction, and sociobiological kinship writ large.

That viruses laterally transfer genes with other viruses and with their hosts thus inculcates them within “cultures of relatedness,” to invoke Carsten (2000) and play on the dual meaning of “culture” to mean both the anthropological concept and the intentional growing of biological materials (like microbes) in Petri dishes. As I argue in this article, replication and reproduction do not define the life of a virus because viruses socially interact with one another in ways that prove that they are life-forms (or living processes), and so lateral gene transfer displaces vertical inheritance and reproduction as a chief hallmark of life.

Sociovirologists are not only challenging and queering biological understandings of viruses as nonliving, individual entities, but, as sociovirologists themselves acknowledge (Rosenbaum, 2019; Zimmer, 2024), rewriting the very field of virology and, perhaps, biology itself. While lateral gene transfer is not a novel discovery of sociovirologists, microbiologists Iris Irby and Sam Brown (2023) contend that Leeks and colleagues (2023) rely on viruses that horizontally transfer genetic material in a destructive manner to make their point about the importance of attending to the social life of viruses. Irby and Brown (2023) suggest that in addition to horizontal transfer, sociovirology would benefit from attention to vertical inheritance in viruses, demonstrating that horizontal gene transfer may be the norm in sociovirological studies, and if viruses are alive, then horizontal gene transfer is a mechanism of aliveness.^15^ As such, horizontal gene transfer may itself be a queer process that displaces reproduction and vertical inheritance, as sociovirology has made this process a cornerstone in its rather queer science.

Returning to evolution is important in this queering process as sociovirologists are also queering temporality, what we might term “reproductive time” or chrononormativity (Freeman, 2010; Halberstam, 2005), by attending to evolutionary time. Queer theorist Elizabeth Freeman (2010,p. xxii, p. 3) defines chrononormativity as “the interlocking temporal schemes necessary for genealogies of descent and for the mundane workings of domestic life,” and “the use of time to organize individual human bodies toward maximum productivity.” Similarly, anthropologist Tom Boellstorff (2007, p. 237) defines “straight time” through “its strict linearity, its evolutionary and millenarian connotations, and its paranoid relationship to futurity.” On the surface, then, sociovirology’s emphasis on (social) evolution seems to reify straight time—there appears to be nothing all that queer about this science or evolutionary time. This is further evidenced by the continued emphasis in queer theory on short-term temporal experiences of the present or not-yet-realized future (Edelman, 2004; Gitzen, 2025a; Lothian, 2018; Muñoz, 2009). Even the slowing of temporality emphasizes a durational, moment-to-moment conception of time (Fishzon, 2017; Gitzen, 2025a; Israeli-Nevo, 2017). Queer studies scholar Jarred Martin (2022, p. 418) might refer to this as “slowtime” or “an affectively intense multi-temporal ‘thick time’ …charged by erotic relations through which new durational qualities of connections (e)merge and are enfolded with/in the human and non-human subjectivities, bodies and forces.”

However, if queer time primarily displaces the role of reproduction within temporal experiences, challenging the capitalist march towards progress alongside individual (species) modes of vertical descent, then I contend it need not solely be about immediacy, be that the radical present or the queer utopic imagining of the future.^16^ The experience of slowtime proves useful, for while Martin (2022) is writing of slow queer practices that extend the present moment^17^—that taking one’s time elongates both the practice and the moment—slowness also indexes the mundane experience of time vis-à-vis evolution. Humans, for instance, cannot rationally feel or experience evolution; we are limited to our lifetimes, and evolution, by human experiential standards, is incredibly slow. Microbes, viruses included, can evolve much quicker than any other life-forms on Earth, and so while by human experiential standards viral evolution may be quick, if we momentarily imagine ourselves as an individual virus we would be met with the slow crawl of evolution. In other words, the slowtime of evolution requires attention to the longue durée immanent to evolution itself.

Queer time, I argue, can capture both the human and viral temporal experience because it challenges normative time-keeping mechanisms and normative experiences of time passing found in (sexual) reproduction. It is important to remember that the conception of queer time originally emerged from human temporal experiences, rooted in those experiences of queer individuals,^18^ but if we broaden queer time to not only the non-human (i.e., virus) but ecological mechanisms (i.e., evolution), then we are not beholden to the immediacy or slowness of the present or not-yet-realized future. Rather, the queer time of viruses lies in both the displacement of (sexual) reproduction within viral lifecycles—not to mention, a chief hallmark of life—including an emphasis on lateral gene transfer, and the importance of virus-virus interactions for long-term evolutionary development. Queer time thus enables a reading of sociovirology as a generational science rooted in social relations rather than descent, whereby life itself emerges over generations—over evolutionary trajectories, developments, and offshoots—rather than located solely within the individual.^19^

A turn to feminist theory proves useful in explicating further my viral understanding of temporality. Feminist philosopher Elizabeth Grosz opines that the field of feminism could do with a bit of Darwinism to recenter questions of not only temporality, but ontology and mattering as well. This begins with evolution, for as Grosz (1999, pp. 34–35) explains, Darwin claimed that the evolution of the species can be explained by attending to three “basic and closely linked principles”: individual variation, proliferation of species and individuals, and natural selection. As Grosz writes, “the evolution of life is possible only through the irreversible temporality of genealogy, which requires an abundance of variation, mechanisms of indefinite, serial or recursive replication/reproduction, and criteria for the selection of differential fitness.” Grosz (2004, p. 5) later writes that “time inhabits all living beings, is an internal, indeed constitutive feature of life itself, yet it is also what places living beings in relations of simultaneity and succession with each other insofar as they are all participants in a single temporality, in a single relentless movement forward.” Darwin provides movement, descent, becoming: “For Darwin, life is essentially linked to the movement of time…being is transformed into becoming, essence into existence, and the past and the present are rendered provisional in light of the force of the future” (Grosz, 2004, p. 7).

Elsewhere I explore the ways that viral relationalities constitute an “allochronic assemblage of potentiality” (Gitzen, 2025b; Prasse-Freeman, 2025), meaning that their suspension in ice and permafrost render them embodiments of that which they were (ancient), that which they are (frozen), and that which they can be (infectious, threatening, and yet potentially beneficial). Viruses are characterized not by their particles but their processes, their co-opting of cells as “viral factories” to produce viral particles, or virions, what we typically “see” or imagine when we think of viruses (Forterre, 2016). Frozen ancient viruses prove useful in explicating a general understanding of viruses given that they embody a simultaneity of temporality—past, present, and future—that queers reproductive time or the temporality of the individual viral particle in favor of an evolutionary time that is in motion, predicated on becoming virus.

In Grosz’s (1999, 2004) estimation, reproduction and replication are important for life: the proliferation of the species and individual are quintessential to species evolution. Yet Grosz does not suggest that life is predicated on the individual’s replication, only that individuals, in general, must proliferate for the species to proliferate. What if life were marked not by the proliferation of the individual viral particle but the longue durée of replication over time, meaning that an altruistic virus may not replicate—the cheater replicates instead—but the survival of the species over time is what marks the virus itself as living? In other words, the virus is alive because it is socially integrated into a larger species assemblage, whereby the individual virus is marked by its contribution to the species rather than its individual potential to replicate. Recalling Griffiths (2015, p. 38), biological and developmental individuality does not actually exist—in a queer theory of/for lichens, we are all lichens because of our symbiotic enmeshment, especially with the microbial ecology living in and through our bodies.

Viruses are living organisms given that their sociality with other viruses contributes to the perpetuation of the species and its subsequent evolution. As such, their aliveness is predicated on evolutionary temporality, not individual or reproductive time, because while the individual, altruistic virus may not survive and thus have “no future” (Edelman, 2004), the species still evolves over a long period of time (or can mutate quite rapidly). The contribution of altruistic viruses to species evolution thus sidesteps the importance of immediate replication in favor of a species-wide, evolutionary temporality that sees individual viruses as part of an allochronic assemblage of potentiality. Stated alternatively, this virus has the potential to individually replicate or not, but such individual replication does not mark the virus as living; rather, their social contribution to evolution—their sociality writ large—is the indicator of aliveness.

## Bodily activity, new stories

In discussing Darwin and humanism, Grosz (2011, p. 22) writes, “each species, each bodily form, orients the world, and its actions in it, according to its ability to maximize action in the world, the kinds of actions that its particular evolved bodily form enables. Life must be understood as the ongoing exploration of and experimentation with the forms of bodily activity that living things are capable of undertaking.” All viruses seem to explore and experiment “with the forms of bodily activity” that they have been bestowed by evolution and natural selection. The seeming incompleteness of cheating VSV—they cannot suppress immune response and instead cause interferon production—means that the cheating VSV is experimenting with a different form of bodily activity, one that often plays in their favor. Altruistic viruses that seem to sacrifice themselves so that genetic relatives, or kin, can replicate and pass on their genetic material are also experimenting with their bodily activities. These viruses do not follow the assumed selfishness and independence of the pathogenic virus because both cheats and cooperators rely on viral sociality and these social behaviors.

Indeed, sociovirology tells a different story about viruses, one that displaces commonly held assumptions of their pathogenic and nonliving qualities, that they are individualistic and exist only to infect. The story that sociovirology tells is one filled with sociality and relationality, where horizontal gene transfer engenders new kinds of relatedness between viruses and between viruses and hosts while viruses cooperate, sacrifice themselves, or cheat their way into replication and natural selection. My argument has not been that reproduction and replication do not matter, but rather that sociovirology displaces the primacy of reproduction and vertical inheritance in the classification of organisms as living. This is a radical position to take, for both sociovirologists and myself, not least of all because for me, most of my biologist interlocutors classify viruses as nonliving.

I am reminded, however, of Villarreal’s (2008) point that perhaps it matters less if viruses are alive or not but rather that viruses play an active role in the evolution of life itself. I agree that it *does* matter that viruses play an active role in the evolution of life, but to claim or even imply viruses as life-forms or living processes is to not only challenge mainstream biology, but I argue, challenge the importance of heteronormative reproduction and vertical inheritance altogether. That, I think, is the more profound social implication, for in challenging heteronormative reproduction and vertical inheritance, sociovirologists are enabling alternative forms of kin making and relatedness to matter in the makeup and evolution of life.

## Footnotes

1 Queer and trans theorist Hil Malatino’s (2019, p. 2) discussion of intersexuality helps to contextualize this discussion, whereby queer corporealities denote “bodies that don’t cohere according to cis-centric, sexually dimorphic, ableist conceptions of comatic normalcy.”

2 I thank Reviewer One for their keen observation with regards to this point.

3 Koonin and colleagues do not necessarily identify as sociovirologists.

4 As a trained anthropologist, such sociocultural depth is necessary to help contextualize knowledge and how it is produced.

5 Elsewhere, I discuss frozen Arctic viruses and bring scientific papers, science writing, and science and technology studies writing in conversation with one another in a similar manner to the ways I do in this article (Gitzen 2025b).

6 The virocell is “a virus-infected cell that will not divide anymore but produces virions,” or virus particles (Forterre 2016, p. 104).

7 Bamford (2003, p. 235) also writes, “The idea of virus lineages would also mean that viruses were present before the separation of the domains of life at the very root of the tree of cellular life.”

8 As Helmreich (2009, p. 82) also notes, “a universal, rooted tree relating all organisms cannot feasibly be placed in corroborating exterior context, since this would entail specifying the initial project of inquiry: all organisms and their relationships.”

9 Clare also recognizes that mainstream epigenetics is limited in its ability to challenge racist, cisgender heterosexist, and classist assumptions about the ways genes and environment can explain relatedness and human behavior. Following Clare, it is important that when queering the notion of kinship via epigenetics that we challenge those very assumptions. I thank Reviewer One for raising this point.

10 It is important to acknowledge that transecological scholars have contributed greatly to work on multispecies relations and around the concepts of time, kinship, and toxicity. See particularly Anita Simah and Banu Subramaniam (2024), Stephanie Clare and Maxine Savage (2024), and Wibke Straube (2024).

11 See Aaron Bradshaw (2023) for greater attention to horizontal or lateral gene transfer in bacteria.

12 Bates (2019, p. 30) similarly implies the queerness or even liberatory nature of evolutionary theory, noting how “some suffragettes used evolutionary theory to challenge the assumption of women’s subordination.” This is not to say that social Darwinists, for instance, did not mobilize evolutionary theory for eugenicist purposes, but that evolutionary theory may lend itself to nonessentialist or even antiessentialist perspectives.

13 As Leeks (2025) writes on his website, “These are small meetings of 50–75 participants, in which we bring together virologists, evolutionary biologists, and other researchers who bring diverse perspectives to the study of virus-virus interactions.”

14 We can trace this observation, that society is like a living organism, back to Émile Durkheim’s (2013) conception of “organic solidarity” to explain how society operates like a biological entity.

15 I recognize, however, that some of the other sociovirological studies discussed above do seem to be writing about vertical inheritance (e.g., Díaz-Muñoz et al., 2017; Leeks and West 2019; Leeks et al., 2021).

16 See also Vanbasten de Araújo and Nicole Seymour’s (2024) contribution to resituate queer/trans time vis-à-vis an archeology of plastic and the longevity of plastic and environmental degradation (and climate change).

17 In particular Martin (2022) is writing of the queer practice of anal fisting among gay men in South Africa.

18 Queer and trans theorist Jack Halberstam (2005), for instance, roots their understanding of queer time in the AIDS crisis of the 1980s and 1990s and the experience of watching gay men die.

19 My invocation of generations is informed by queer Indigenous time that incorporates past ancestors and future relations of care into understandings and experiences of time (Spice 2024).
